# RETRACTION: Ameliorating Effect of *Malva neglecta* Wallr on Obesity and Diabetes in Wistar Rats: A Mechanistic Study

**DOI:** 10.1155/bmri/9879623

**Published:** 2025-10-26

**Authors:** BioMed Research International

RETRACTION: M. Furqan Akhtar, U. Farooq, A. Saleem, M. Saleem, M. Habibur Rahman, and G. M. Ashraf, “Ameliorating Effect of *Malva neglecta* Wallr on Obesity and Diabetes in Wistar Rats: A Mechanistic Study,” *BioMed Research International* 2022 (2022): 2614599. 10.1155/2022/2614599.

The above article, published online on 28 May 2022 in Wiley Online Library (http://wileyonlinelibrary.com), has been retracted following the agreement of the journal’s Section Editor, Jinsong Ren; and John Wiley & Sons Ltd.

The retraction has been agreed following an investigation of the concerns raised by *Mycosphaerella arachidis* on PubPeer [1], which identified unexpected similarities between multiple panels of Figures 3 and 4.

More specifically, the images in the panels a b, c, and e of Figure 3 share overlapping features, despite representing animals from different treatment groups. Further concerns have been identified related to the similarity of images shown in Figure 4d and Figure 4e.

As a result of the investigation, the data and conclusions of this article are considered unreliable.

Muhammad Furqan Akhtar and Ammara Saleem and disagree with this retraction. The other authors have been informed of this decision but did not provide a response.
